# Improving Predictions of Protein-Protein Interfaces by Combining Amino Acid-Specific Classifiers Based on Structural and Physicochemical Descriptors with Their Weighted Neighbor Averages

**DOI:** 10.1371/journal.pone.0087107

**Published:** 2014-01-28

**Authors:** Fábio R. de Moraes, Izabella A. P. Neshich, Ivan Mazoni, Inácio H. Yano, José G. C. Pereira, José A. Salim, José G. Jardine, Goran Neshich

**Affiliations:** 1 Biology Institute, University of Campinas, Campinas, São Paulo, Brazil; 2 Brazilian Agricultural Research Corporation (EMBRAPA), National Center for Agricultural Informatics, Campinas, São Paulo, Brazil; 3 School of Electrical and Computer Engineering, University of Campinas, Campinas, São Paulo, Brazil; CSIR-Institute of Microbial Technology, India

## Abstract

Protein-protein interactions are involved in nearly all regulatory processes in the cell and are considered one of the most important issues in molecular biology and pharmaceutical sciences but are still not fully understood. Structural and computational biology contributed greatly to the elucidation of the mechanism of protein interactions. In this paper, we present a collection of the physicochemical and structural characteristics that distinguish interface-forming residues (IFR) from free surface residues (FSR). We formulated a linear discriminative analysis (LDA) classifier to assess whether chosen descriptors from the BlueStar STING database (http://www.cbi.cnptia.embrapa.br/SMS/) are suitable for such a task. Receiver operating characteristic (ROC) analysis indicates that the particular physicochemical and structural descriptors used for building the linear classifier perform much better than a random classifier and in fact, successfully outperform some of the previously published procedures, whose performance indicators were recently compared by other research groups. The results presented here show that the selected set of descriptors can be utilized to predict IFRs, even when homologue proteins are missing (particularly important for orphan proteins where no homologue is available for comparative analysis/indication) or, when certain conformational changes accompany interface formation. The development of amino acid type specific classifiers is shown to increase IFR classification performance. Also, we found that the addition of an amino acid conservation attribute did not improve the classification prediction. This result indicates that the increase in predictive power associated with amino acid conservation is exhausted by adequate use of an extensive list of independent physicochemical and structural parameters that, by themselves, fully describe the nano-environment at protein-protein interfaces. The IFR classifier developed in this study is now integrated into the BlueStar STING suite of programs. Consequently, the prediction of protein-protein interfaces for all proteins available in the PDB is possible through STING_interfaces module, accessible at the following website: (http://www.cbi.cnptia.embrapa.br/SMS/predictions/index.html).

## Introduction

Protein-protein interactions are very specific in the sense that they control almost all processes within cells, such as signal transduction, metabolic and gene regulation, and immunologic responses. [1]. Notably, even in a crowded intracellular environment [2], each one of these distinct protein-protein interactions is mediated through a particular area of the protein surfaces [1].

To detect protein-protein interactions with resolution ranging from the cellular to the atomic level, many experimental methods may be employed [3]. Nevertheless, the combination of experiments, which should offer a more detailed understanding of the protein interaction network, usually takes a long time, especially during protein sample preparation. Such a bottleneck of current experimental techniques makes *in silico* approaches for characterizing macromolecular complexes very useful as these approaches may guide *in vivo* and *in vitro* experiments, reducing temporal and financial costs.

However, similar to some experimental techniques used to gather information about protein interfaces (for example, obtaining 3D protein structures through x-ray diffraction or nuclear magnetic resonance techniques), computational methods also do face some challenges. These difficulties include predicting quaternary structure via template-based docking algorithms, which can only yield atomic details of protein-protein interactions if the sequence identity to another known protein complex structure is higher than approximately 60%. If the sequence homology to known protein complexes falls between 30% and 60%, structural similarity is conserved but details, such as residue pairing, are not predicted correctly. When the similarity is less than 30%, no reliable model is obtained and only the relative orientation of the molecules is predicted [4]. Recent reviews on protein docking methods have emphasized this issue in details [46]–[48]. Therefore, a more accurate understanding of the principal amino acid characteristics in protein-protein interfaces is required to improve the quality and reliability of *in silico* generated protein complex models. An advanced knowledge of this particular location may result in much better structure predictions of the entire complex. This improvement is mainly because all protein-protein interactions occur only at a portion of the protein surface: the interface between the molecules. In fact, it has been argued that monomeric subunits have all the necessary features for establishing protein-protein interactions [1].

Thus, it is not surprising that Schneider and colleagues [9] approached the feasibility of predicting protein-protein binding sites even when no interacting partner is present/known. Accordingly, the information required for establishing an interaction with another protein is already present in the tridimensional structure of a single protein. In any case, one cannot ignore the fact that the characteristics of the protein environment also can play an important role by being able to modify protein structures and, consequently, interfaces. Additionally, it is also clear that if a protein is interacting with two or more different partners, different interfaces may be formed for each partner.

A careful literature review will quickly confirm that although there are several recently published studies regarding the characteristics that could determine differences between interface-forming residues (IFR) and free surface residues (FSR) [1], [5]–[8], there is no general agreement about exactly how proteins associate with each other and which descriptors of their characteristics are suitable for elucidating this mechanism [49]–[54]. Also, by comparing the interface area against the rest of the free surface is a common procedure during attempts to characterize the main differences between those two classes. This type of comparison has been described in recent studies, including those cited above.

A variety of models and descriptors were explored to build protein-protein interface classifiers. Promate [9] and PINUP [22] used linear scoring functions, while PPI-Pred [25] used a support vector machine approach, SPPIDER [23] and cons-PPISP [26] used a neural network model, and Meta-PPISP combined the results of cons-PPISP, Promate and PINUP as a meta-predictor. In contrast to the method proposed in this study, the six mentioned models make use of amino acid sequence conservation and propensity. How important is this difference among Sting-LDA and other mentioned algorithms could only be accessed adequately if proper analysis is done on how often the conservation property could not be used in known protein (sequence and structure) universe. It is known that structural genome projects used high-throughput techniques [56], [57], [58] to produce and then deposit in the PDB thousands of new structures. For instance, half of the protein structures solved during the year of 2005 came from structural genome initiatives, including structures of the so-called orphan proteins. Orphan proteins are organism-specific proteins, i.e., they have no homologue protein in other lineages. Estimates are that up to one third of the genes/proteins from whole known genomes accounts for orphan proteins [59]. Ekman *et al.* [60] show, using the structural classification of protein (SCOP) [61], that up to 25% of the known non-redundant protein structures from bacteria are from orphan proteins or from proteins having an *orphan domain*. Also, up to 21% of known protein structures in Eukarya kingdom and 24% in Archaea kingdom follow the same trend. Operating in such scenario where limitations imposed by orphan protein existence restricts the use of aforementioned algorithms dependent on conservation parameter for predicting interface residues, would clearly lead to unreliable results. Therefore, the strong demand is created for the development of more general approaches for IFR prediction which would have similar performance to conservation dependent algorithms, yet without the use of evolutionary-related attributes for prediction. The Sting-LDA was produced having in mind this demand as well.

We report results on the classification of the 20 naturally occurring amino acids into two distinct classes: IFR and FSR, by using several amino acid descriptors from the BlueStar STING database [10]–[13]. BlueStar STING has been used previously for predicting enzyme class [14], protein-ligand analysis [15], [16], protein mutant analysis [17], [18], and protein-protein interaction pattern analysis [19], mostly because BlueStar STING offers easy access to a very rich repository of protein characteristics. The analysis reported in this paper uses only the information in the three-dimensional structure for predicting protein interfaces. Thus, sequence conservation attributes are not explicitly used because inferences embedded into them are mere consequences of preserving the nano-environment (which by itself dictates a particular function for a specific amino acid ensemble (such as a catalytic site or an interface)). Therefore, our method still functions in scenarios in which no close homologue to a protein of interest is available and in which all other methods that rely on the use of “conservation” attributes would fail. Nevertheless, for a complete assessment of the suitability of the selected physicochemical and structural descriptors for classifying amino acid residues as interface residues and also of the importance of the contribution of sequence conservation attributes to the same classification, an additional IFR classifier was built that also includes the “*amino acid conservation*” attribute from STING database [20]. We show that all the clues necessary for IFR prediction may already be contained within the information in descriptors that are derived only from the protein structure. Furthermore, the addition of “conservation” does not improve the performance indicators for IFR classification if an information plateau with a sufficiently high number of structural attributes is reached that completely describes the nano-environment of the interface. Additionally, we describe (for the first time) how to construct and use classifiers that are specific to amino acid type for additional improvements in predicting IFRs.

## Results and Discussion

### Linear correlation among attributes

A long list of amino acid descriptors from the STING database (table 1 lists all selected descriptors and their variations included in this study) was subjected to analysis to determine whether there is a linear correlation among them. The existence of non-orthogonal attributes present in table 1 may be illustrated, for example, by analyzing the case of “*Sting hydrophobicity*”, which is calculated by multiplying the relative accessibility of an amino acid with its hydrophobicity index (from the Radzicka scale [21]). Therefore, the descriptors “*accessibility”*, “*relative accessibility”* and “*Sting hydrophobicity*” are all correlated for the same amino acid type (e.g., for all alanine residues) but uncorrelated among different amino acid types. Consequently, only the “*Sting hydrophobicity*” descriptor was used for further analysis, while “*accessibility”* and “*relative accessibility”* were eliminated for redundancy. Table 2 summarizes the results from determining the orthogonality of other structural descriptors. The “x” in table 2 refers to removed descriptors (because of identified linear correlations with some other attributes), while the “-” sign is assigned to attributes that are not applicable to specific amino acids. From table 2, we can observe that the attributes *Contact Energy Density (CED) at the Cα (6Å)*, *CED at Last Heavy Atom (LHA) (5Å)*, *CED at LHA (6Å)*, *density at Cα (6Å)*, *density at LHA (6Å)*, *sponge at Cα (6Å)*, *sponge at LHA (6Å)*, *electrostatic potential-average* and *Cross Link Order at Cβ* are linearly correlated with another variation of the same descriptor in most amino acid types.

**Table 1:** Selected Descriptors from the BlueStar STING database that were used for IFR prediction.

**Accessibility – Acc**						
	Acc in isolation			Relative Acc		
**Eletrostatic Potential – EP**						
EP @ Cα	EP @LHA		EP average	EP @ surface		
**Hydrophobicity**						
Hydrophobicity in isolation						
**Contact Energy Density – CED/Internal (INT) contacts**						
CED @ Cα INT (3)	CED @ Cα INT (6)		CED @ LHA INT (3)	CED @ LHA INT (6)		
CED @ Cα INT (4)	CED @ Cα INT (7)		CED @ LHA INT (4)	CED @ LHA INT (7)		
CED @ Cα INT (5)			CED @ LHA INT (5)			
**Cross Link Order (CLO)**						
CLO @ Cα			CLO @ Cβ			CLO @ LHA
**Cross Presence Order (CPO)**						
CPO @ Cα			CPO @ Cβ			CPO @ LHA
**Dihedral angles**						
	PHI			PSI		
**Rotamers**						
CHI 1	CHI 2		CHI 3	CHI 4		
**Density**						
Density @ Cα (3)	Density @ Cα (6)		Density @ LHA (3)	Density @ LHA (6)		
Density @ Cα (4)	Density @ Cα (7)		Density @ LHA (4)	Density @ LHA (7)		
Density @ Cα (5)			Density @ LHA (5)			
**Sponge**						
Sponge @ Cα (3)		Sponge @ Cα (6)	Sponge @ LHA (3)		Sponge @ LHA (6)	
Sponge @ Cα (4)		Sponge @ Cα (7)	Sponge @ LHA (4)		Sponge @ LHA (7)	
Sponge @ Cα (5)			Sponge @ LHA (5)			
**Contact Energy**						
Hydrophobic contacts	Hydrogen bond - MM		Hydrogen bond - MWM	Hydrogen bond - MWWM		Aromatic contacts
Charged (Attractive)	Hydrogen bond - MS		Hydrogen bond - MWS	Hydrogen bond - MWWS		Disulfide bond
Charged (Repulsive)	Hydrogen bond - SS		Hydrogen bond - SWS	Hydrogen bond - SWWS		Total contact energy
**Unused Contact Energy**						
Hydrophobic contacts	Hydrogen bond - MM		Hydrogen bond - MWM	Hydrogen bond - MWWM		Aromatic contacts
Charged (Attractive)	Hydrogen bond - MS		Hydrogen bond - MWS	Hydrogen bond - MWWS		Disulfide bond
Charged (Repulsive)	Hydrogen bond - SS		Hydrogen bond - SWS	Hydrogen bond - SWWS		Total contact energy

**Labels**: Cα  =  Carbon alpha; LHA  =  Last heavy atom; MM  =  Main chain – Main chain; MS  =  Main chain – Side chain; SS  =  Side chain – Side chain; W  =  Water molecule; WW  =  2x Water molecules.

* A complete description of the listed physicochemical and structural attributes is given at http://www.cbi.cnptia.embrapa.br/SMS and in [10]–[13], [20].

**Table 2:** List of descriptors and variables from the BlueStar STING database that were removed after linear correlation analysis.

ASP	HIS	LEU	CYS	GLU
CED @ Ca (4)	CED @ Ca (5)	CED @ Ca (6)	CED @ Ca (6)	CED @ Ca (5)
CED @ Ca (5)	CED @ Ca (6)	CED @ LHA (4)	CED @ LHA (4)	CED @ Ca (6)
CED @ Ca (6)	CED @ LHA (4)	CED @ LHA (5)	CED @ LHA (5)	CED @ LHA (4)
CED @ LHA (4)	CED @ LHA (5)	CED @ LHA (6)	CED @ LHA (6)	CED @ LHA (5)
CED @ LHA (5)	CED @ LHA (6)	CLO atC-beta	CLO atC-beta	CED @ LHA (6)
CED @ LHA (6)	CLO atC-beta	CLO @ LHA	CLO @ LHA	CLO atC-beta
CLO atC-beta	CLO @ LHA	Densityat Ca (4)	Densityat Ca (6)	Densityat Ca (4)
Densityat Ca (5)	Densityat Ca (5)	Densityat Ca (5)	Densityat LHA (5)	Densityat Ca (6)
Densityat LHA (6)	Densityat Ca (6)	Densityat Ca (6)	Densityat LHA (6)	Densityat LHA (6)
EP Average	Densityat LHA (6)	Densityat LHA (6)	EP Average	EP Average
Spongeat Ca (4)	EP Average	EP Average	Spongeat Ca (4)	Spongeat Ca (4)
Spongeat Ca (5)	Spongeat Ca (4)	Spongeat Ca (4)	Spongeat Ca (5)	Spongeat Ca (5)
Spongeat Ca (6)	Spongeat Ca (5)	Spongeat Ca (5)	Spongeat Ca (6)	Spongeat Ca (6)
Spongeat LHA (4)	Spongeat Ca (6)	Spongeat Ca (6)	Spongeat LHA (5)	Spongeat LHA (5)
Spongeat LHA (5)	Spongeat LHA (5)	Spongeat LHA (5)	Spongeat LHA (6)	Spongeat LHA (6)
Spongeat LHA (6)	Spongeat LHA (6)	Spongeat LHA (6)		
ILE	LYS	MET	PHE	PRO
CED @ Ca (6)	CED @ Ca (6)	CED @ Ca (6)	CED @ Ca (6)	CED @ Ca (6)
CED @ LHA (4)	CED @ LHA (4)	CED @ LHA (4)	CED @ LHA (4)	CED @ LHA (4)
CED @ LHA (5)	CED @ LHA (5)	CED @ LHA (5)	CED @ LHA (5)	CED @ LHA (5)
CED @ LHA (6)	CED @ LHA (6)	CED @ LHA (6)	CED @ LHA (6)	CED @ LHA (6)
CLO atC-beta	CLO atC-beta	CLO atC-beta	Densityat Ca (4)	CLO atC-beta
CLO @ LHA	Densityat Ca (4)	Densityat Ca (4)	Densityat Ca (5)	Densityat Ca (4)
Densityat Ca (5)	Densityat Ca (5)	Densityat Ca (5)	Densityat Ca (6)	Densityat Ca (5)
Densityat Ca (6)	Densityat Ca (6)	Densityat Ca (6)	Densityat LHA (6)	Densityat Ca (6)
Densityat LHA (6)	Densityat LHA (6)	Densityat LHA (6)	EP Average	Densityat LHA (6)
EP Average	EP Average	EP Average	Spongeat Ca (4)	EP Average
Spongeat Ca (4)	Spongeat Ca (4)	Spongeat Ca (4)	Spongeat Ca (5)	Spongeat Ca (4)
Spongeat Ca (5)	Spongeat Ca (5)	Spongeat Ca (5)	Spongeat Ca (6)	Spongeat Ca (5)
Spongeat Ca (6)	Spongeat Ca (6)	Spongeat Ca (6)	Spongeat LHA (4)	Spongeat Ca (6)
Spongeat LHA (5)	Spongeat LHA (5)	Spongeat LHA (5)	Spongeat LHA (5)	Spongeat LHA (5)
Spongeat LHA (6)	Spongeat LHA (6)	Spongeat LHA (6)	Spongeat LHA (6)	Spongeat LHA (6)
TYR	VAL	SER	THR	TRP
CED @ Ca (6)	CED @ Ca (6)	CED @ Ca (6)	CED @ Ca (6)	CED @ Ca (6)
CED @ LHA (5)	CED @ LHA (4)	CED @ LHA (6)	CED @ LHA (4)	CED @ LHA (4)
CED @ LHA (6)	CED @ LHA (5)	CLO atC-beta	CED @ LHA (5)	CED @ LHA (5)
CLO atC-beta	CED @ LHA (6)	CLO @ LHA	CED @ LHA (6)	CED @ LHA (6)
Densityat Ca (4)	CLO atC-beta	Densityat Ca (4)	CLO atC-beta	Densityat Ca (5)
Densityat Ca (5)	Densityat Ca (4)	Densityat Ca (5)	Densityat Ca (5)	Densityat Ca (6)
Densityat Ca (6)	Densityat Ca (5)	Densityat Ca (6)	Densityat Ca (6)	Densityat LHA (6)
Densityat LHA (6)	Densityat Ca (6)	Densityat LHA (6)	Densityat LHA (6)	EP Average
EP Average	Densityat LHA (6)	EP Average	EP Average	Spongeat Ca (4)
Spongeat Ca (4)	EP Average	Spongeat Ca (4)	Spongeat Ca (4)	Spongeat Ca (5)
Spongeat Ca (5)	Spongeat Ca (4)	Spongeat Ca (5)	Spongeat Ca (5)	Spongeat Ca (6)
Spongeat Ca (6)	Spongeat Ca (5)	Spongeat Ca (6)	Spongeat Ca (6)	Spongeat LHA (5)
Spongeat LHA (4)	Spongeat Ca (6)	Spongeat LHA (5)	Spongeat LHA (5)	Spongeat LHA (6)
Spongeat LHA (5)	Spongeat LHA (5)	Spongeat LHA (6)	Spongeat LHA (6)	Spongeat LHA (6)
Spongeat LHA (6)	Spongeat LHA (6)			
ALA	ASN	GLN	ARG	GLY
CED @ Ca (6)	CED @ Ca (6)	CED @ Ca (6)	CED @ Ca (6)	CED @ Ca (6)
CED @ LHA (4)	CED @ LHA (6)	CED @ LHA (5)	CED @ LHA (4)	Densityat Ca (5)
CED @ LHA (6)	CLO atC-beta	CED @ LHA (6)	CED @ LHA (5)	Densityat Ca (6)
CLO atC-beta	Densityat Ca (6)	Densityat Ca (5)	CED @ LHA (6)	EP Average
CLO @ LHA	Densityat LHA (6)	Densityat Ca (6)	Densityat Ca (5)	Spongeat Ca (4)
Densityat Ca (5)	EP Average	Densityat LHA (6)	Densityat Ca (6)	Spongeat Ca (5)
Densityat Ca (6)	Spongeat Ca (4)	EP Average	Densityat LHA (6)	Spongeat Ca (6)
Densityat LHA (7)	Spongeat Ca (5)	Spongeat Ca (4)	Spongeat Ca (4)	
EP Average	Spongeat Ca (6)	Spongeat Ca (5)	Spongeat Ca (6)	
Spongeat Ca (5)	Spongeat LHA (4)	Spongeat Ca (6)	Spongeat LHA (5)	
Spongeat Ca (7)	Spongeat LHA (5)	Spongeat LHA (5)	Spongeat LHA (6)	
Spongeat LHA (5)	Spongeat LHA (6)	Spongeat LHA (6)		
Spongeat LHA (6)				

The number within parenthesis (indicated for some descriptors) represents the radius of the probing sphere used to calculate the numerical value of the respective attribute.

The same procedure was applied to the “weighted neighbor averages” descriptors (WNA, see Methods for more details). Table 3 shows the selected WNA descriptors after the removal of linearly correlated descriptors from the initial “weighted neighbor averages” ensemble. The newly created set was composed of selected descriptors from tables 2 and 3. This set was then used in principal component analysis and for building the linear models. These models were formed using linear classifiers via discriminant analysis while ensuring that data redundancy and consequent over-fitting of classifiers was eliminated.

**Table 3:** List of selected “weighted neighbor averages” (WNA) descriptors.

Descriptors	
Density @ ca3 WNADist	UnusedChargedattractive Energy WNASurf
Density @ ca3 WNASurf	UnusedChargedrepulsive Energy WNADist
EP Average WNADist	UnusedChargedrepulsive Energy WNASurf
EP Average WNASurf	UnusedDissulfidebond Energy WNADist
EP @ ca WNADist	UnusedDissulfidebond Energy WNASurf
EP @ ca WNASurf	Unused Hydrogen Bond - MM Energy WNADist
EP @ lha WNADist	Unused Hydrogen Bond - MM Energy WNASurf
EP @ lha WNASurf	Unused Hydrogen Bond - MS Energy WNADist
EP @ Surface WNADist	Unused Hydrogen Bond - MS Energy WNASurf
Energy Density @ ca3 WNADist	Unused Hydrogen Bond - MWM Energy WNADist
Energy Density @ ca3 WNASurf	Unused Hydrogen Bond - MWM Energy WNASurf
Energy Density @ ca4 WNADist	Unused Hydrogen Bond - MWSEnergy WNADist
Energy Density @ ca4 WNASurf	Unused Hydrogen Bond - MWS Energy WNASurf
Energy Density @ ca7 WNASurf	Unused Hydrogen Bond - MWWM Energy WNADist
Energy Density @ lha3 WNADist	Unused Hydrogen Bond - MWWM Energy WNASurf
Energy Density @ lha3 WNASurf	Unused Hydrogen Bond - MWWS Energy WNADist
Hydrophobicity WNADist	Unused Hydrogen Bond - MWWS Energy WNASurf
Sponge @ ca3 WNADist	Unused Hydrogen Bond - SS Energy WNADist
Sponge @ ca3 WNASurf	Unused Hydrogen Bond - SS Energy WNASurf
Sponge @ lha7 WNADist	Unused Hydrogen Bond - SWS Energy WNADist
Sponge @ lha7 WNASurf	Unused Hydrogen Bond - SWS Energy WNASurf
Total Unused Contact Energy WNADist	Unused Hydrogen Bond - SWWS Energy WNADist
UnusedAromaticcontacts Energy WNADist	Unused Hydrogen Bond - SWWS Energy WNASurf
UnusedAromaticcontacts Energy WNASurf	UnusedHydrophobiccontacts Energy WNADist
UnusedChargedattractive Energy WNADist	UnusedHydrophobiccontacts Energy WNASurf

### Principal Component Analysis

The number of orthogonal (independent/non-redundant) attributes (principal components) was selected to form a new set of attributes, which were specific for each amino acid type, by calculating the correlation matrix from a training set of data. The descriptors that had a high linear correlation with other descriptors were eliminated from further use in this procedure - a crucial procedural decision undertaken to avoid posterior model over fitting. The resulting set of descriptors was composed of 22 principal components for glycine with the number of principal components increasing to 37 for tryptophan and arginine. After integrating the WNA descriptors, the number of principal components grew to 28 for glycine and peaked at 41 principal components for arginine. Each specific training set from each cross validation step actually has a different correlation matrix. Extensive calculation revealed that the number of principal components that accounted for 95% of the training set variability (see Methods for further details on selecting the number of principal components) did not fluctuate much. Consequently, the number of principal components for each specific amino acid was maintained at a constant value for all cross-validation steps. However, the different number of principal components for each amino acid type indicates the existence of different eigenvectors and thus a different correlation matrix, i.e., for each amino acid type, the descriptors and their variations correlate differently with each other. This result is a principal motivation for our decision to formulate all classifiers in a “per amino acid” fashion.

### Linear classifiers via discriminant analysis (LDA)

As explained in the Methods section, the dataset (DS30) used in this study was created by selectively retrieving PDB files from the PDB. While the information about “true” IFRs can be easily obtained from specific tridimensional structures, the retrieval of “true” FSRs is not so straightforward. This difficulty is because one cannot be completely sure that a given free surface residue will not be part of a completely different interface formed with another partner (perhaps one that has not been co-crystallized yet) [49]. Taking that bias into consideration, only information available in the original PDB structure was used.

**Figure 1:**
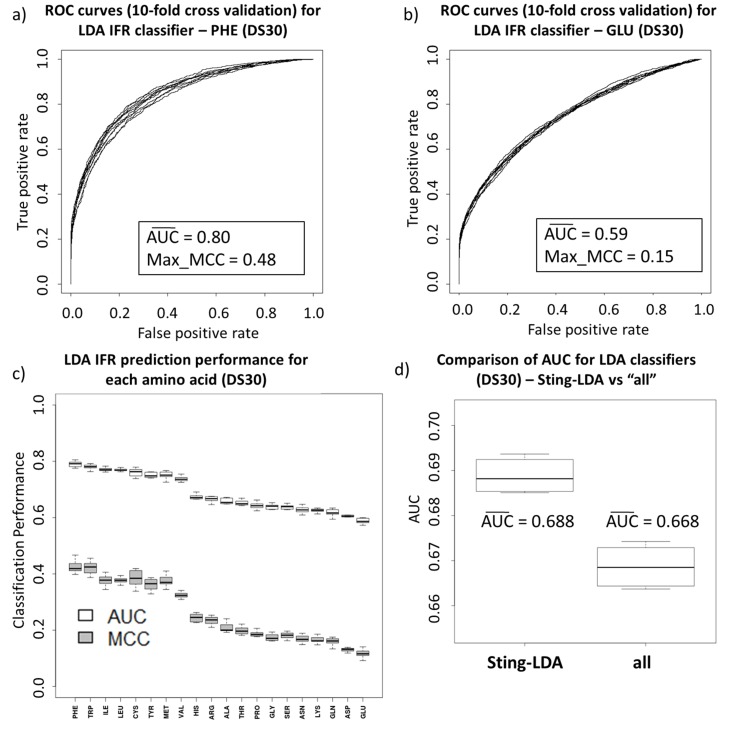
Comparing LDA classifiers with ROC analysis. Performance evaluation using ROC analysis for the tryptophan LDA classifier (a), glycine LDA classifier (b) and the aggregated result (in gray) of the 20 independent amino acid LDA classifiers (d). In blue, 10 ROC curves (from 10-fold cross validation tests) are presented for the classifier that is not specific to the type of amino acid. Ten-fold cross validation was used, and the performance indicators AUC and MCC are displayed for both classifiers in (a) and (b). The results for all generated classifiers are shown in (c): the AUC (white) and MCC (gray). Tryptophan serves as the best classifier but is closely followed by (in AUC criteria) aspartic acid, methionine, isoleucine, leucine and valine. The Sting-LDA aggregated classifier, which uses 20 amino acid-specific classifiers with no WNA descriptors, has an AUC average of 0.828, whereas the amino acid-unspecific classifier has an AUC average of 0.751.

In addition, whenever a protein was available in more than one PDB structure file with different partners and, potentially, different interface regions, our selection algorithm only kept one example (the first ranked PDB cluster) of the common (“base”) protein chain. The other IFRs on the common protein chain, built by the discharged chain, were not taken into account. Although we could use a mapping procedure, similar to previously described methods such as SPPIDER [23], we could not determine with certainty how the lack of including differences in the nano-environments of the considered interfaces (through use of corresponding amino acid characteristics descriptors) would influence the results. Therefore, we opted not to use any mapping of the “missed” IFRs on the unchanged “base” structures. Nevertheless, our structure selection algorithm retained all of the non-redundant chains. So, the IFRs from partners to a “base” protein were included in the used dataset and, therefore, the “missed” IFRs on the “base” protein were indirectly present, as long as sequence of partner protein did not share more 30% sequence similarity with any other protein chain in the DS30. The 20 amino acid-specific linear classifiers obtained via discriminant analysis (no “weighted neighbor averages” descriptors used in the first stage) were subjected to receiver operating characteristic (ROC) evaluation. In figure 1, we present the ROC curves for the 10-fold cross validation of the tryptophan classifier (1-a) and for the glycine classifier (1-b). The tryptophan LDA classifier is the best overall when considering the area under the ROC curve (AUC) and the maximum Matthew’s correlation coefficient (MCC) criteria. In contrast, the glycine results indicate that this particular amino acid had the lowest performance rates among all amino acids for distinguishing IFRs from FSRs. Nevertheless, the glycine LDA classifier outperformed a random classifier (for which AUC  =  0.5 and MCC  =  0), clearly showing that the use of physicochemical and structural descriptors helps in predicting IFRs and FSRs. Figure 1-c summarizes the results of the 10-fold cross-validation for all amino acid-specific classifiers, sorted by average AUC. As mentioned above, the tryptophan LDA classifier has the best AUC and Matthew’s correlation coefficient rates, closely followed by the aspartic acid, isoleucine, leucine, cysteine and valine classifiers. When aggregating the 20 amino acid-specific classifiers (referred to in figure 1 as Sting-LDA), an average AUC value equal to the average of the LDA amino acid-specific classifiers was achieved (figure 1-d). We also generated an LDA classifier with no distinction among amino acid types. It is important to observe the clear performance gain resulting from dividing the dataset per residue fashion and then formulating amino acid-specific classifiers. This procedure resulted in an AUC increase from 0.751 to 0.828. By using Welch’s two-sample statistical testing [14], we were assured that the performance difference between the amino acid-specific and the unspecific approach was statistically significant, with a *p-value* of 10^−10^.

**Figure 2:**
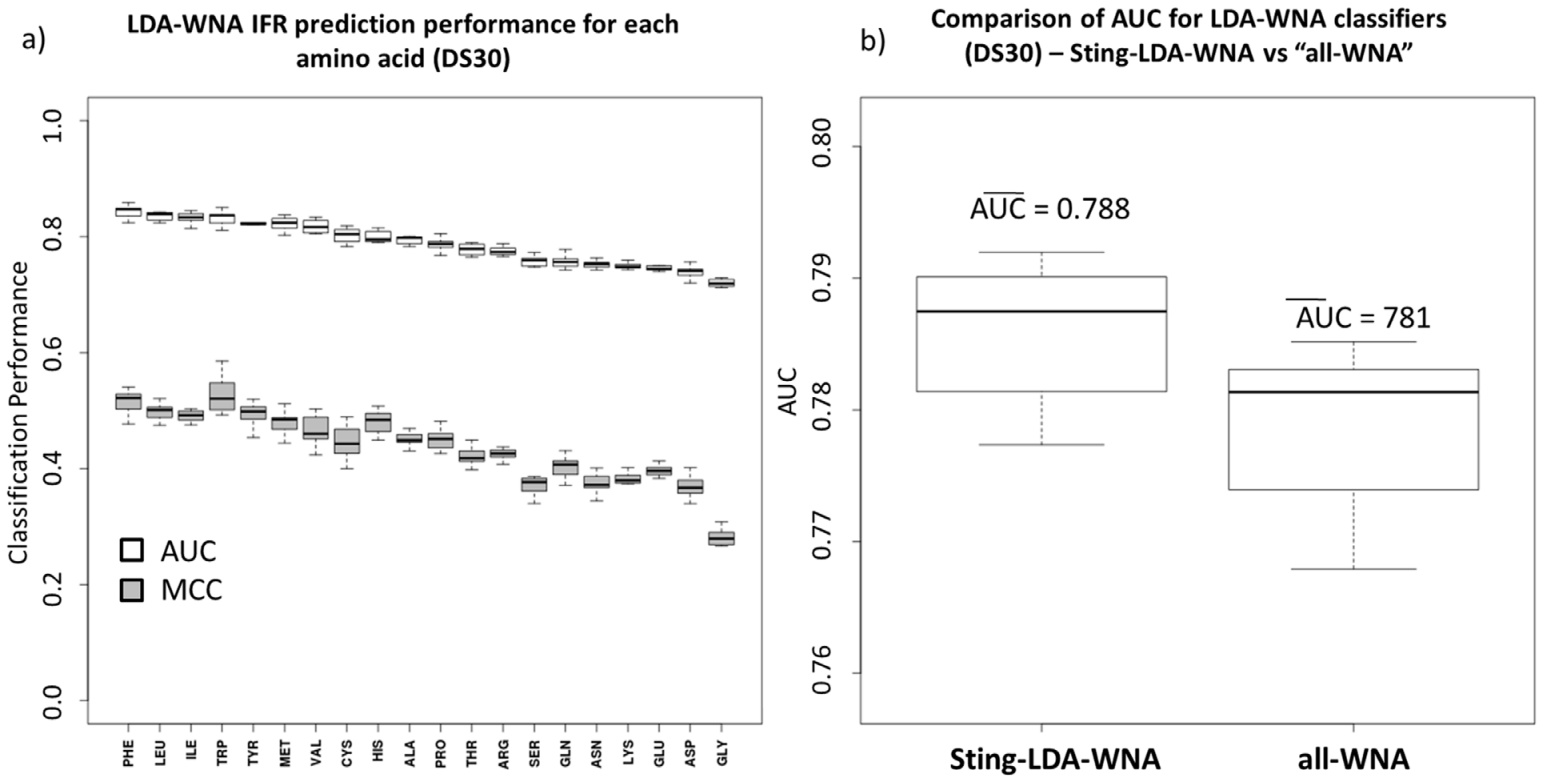
Comparing LDA classifiers using weighted neighbor averages (WNA) descriptors with ROC analysis. The results for all generated amino acid classifiers are shown in (a): the AUC (white) and MCC (gray). The amino acid ranking order is similar to that of figure 1-c, except for glycine and cysteine LDA models. The Sting-LDA-WNA aggregated classifier from the 20 amino acid-specific classifiers with WNA descriptors has an AUC average of 0.949, whereas the amino acid-unspecific classifier average was 0.944 (indicating that the performance gain while using the formulated amino acid-specific classifiers is statistically relevant and, therefore, recommended for better IFR classification).

Next, the addition of “weighted neighbor averages” descriptors remarkably increased the classification performance for all amino acid-specific classifiers as well as for the amino acid-unspecific classifier (figure 2). The amino acid order (with respect to increasing AUC), shown in figure 2-a, changed when compared with figure 1-c. The tryptophan and aspartic acid LDA classifiers are still the best classifier models using both the AUC and Matthew’s correlation coefficient criteria. Glycine improved in ranking by many positions in this ordered list to become the 8^th^ ranked amino acid. The opposite behavior was observed for the cysteine classifier, which became the 15^th^ ranked amino acid in figure 2-a.

As mentioned above, the performance gain, upon formulating amino acid-specific classifiers for the case in which “weighted neighbor averages” descriptors were present, is observed (figure 2-b). However, the aggregated 20 amino acid-specific classifier, with “weighted neighbor averages” descriptors (referred to as Sting-LDA-WNA), is still slightly better than the amino acid-unspecific classifier with WNA descriptors. Welch’s two-sample statistical testing [14] for this particular case results in a *p-value* of approximately 10^−8^, indicating a statistically significant gain in performance for the aggregated specific classifier

**Figure 3:**
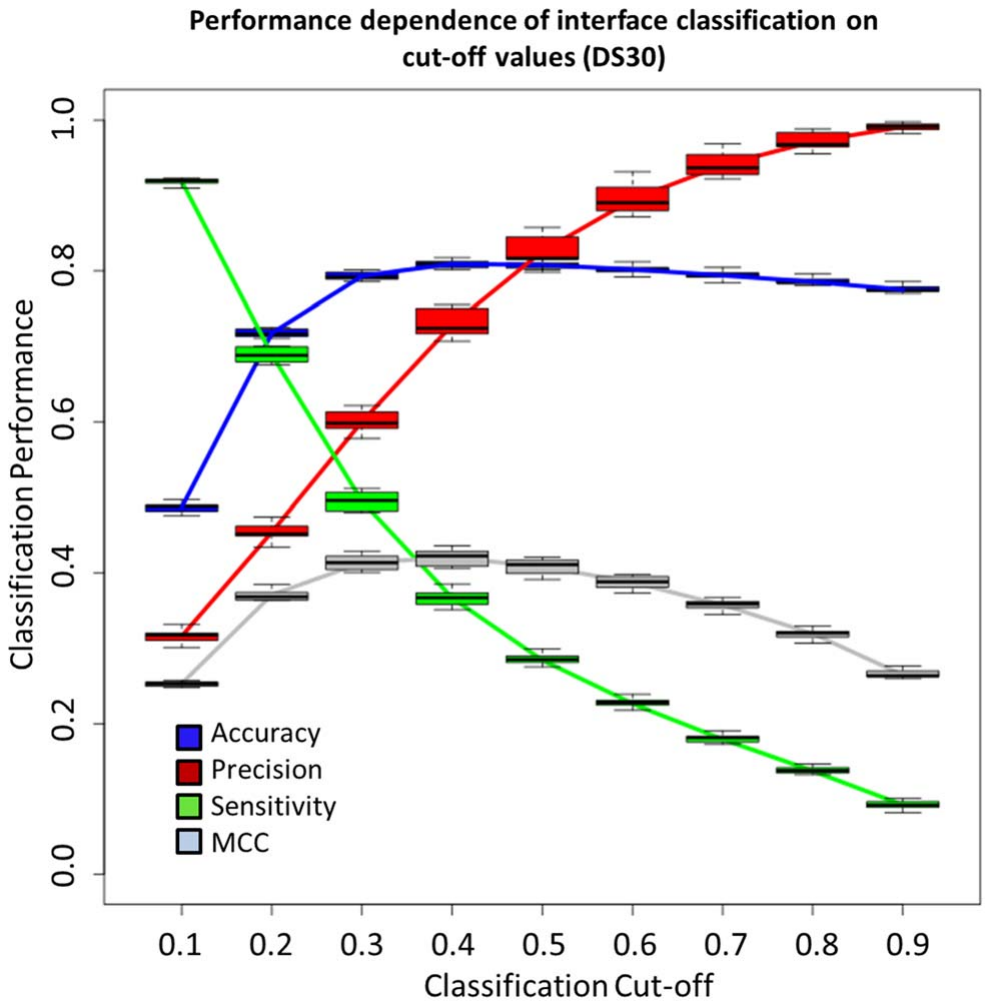
Cut-off performance dependence of Sting-LDA-WNA classifier. The performance indicators observed for the classifiers built from DS30. When the cut-off is increased from 0.10 to 0.50, the accuracy increases and reaches its peak, and then it gradually decreases with a further increase in the cut-off value. The precision rate only grows as the cut-off is increased, that is, for higher cut-off values, fewer entries are classified as IFR, leading to more false positives. For sensitivity, the opposite behavior is observed (illustrating the performance trade-off). When increasing the cut-off, more entries are labeled as FSR, and fewer labeled as IFR are misclassified. The highest MCC value occurred when using the same cut-off as that for the highest accuracy, which is 0.50. Box plots were obtained with 10-fold cross validation.

Because of its improved classification performance, we used the Sting-LDA-WNA classifier to continue the analysis in this study as well as for comparison with other available methods in the literature. Using 10-fold cross-validation and screening the classification cut-off values (ranging from 0.1 to 0.9), the classification limits of Sting-LDA-WNA were tested. Figure 3 shows the performance change for 9 of the tested cut-off values. The precision and sensitivity rates have contrasting behavior as the cut-off value increases: while the precision grows, the sensitivity decreases. The best accuracy and Matthew’s correlation coefficient occur when using a cut-off value of 0.50. This value appears to be the most appropriate cut-off for general purposes, unless the precision rate is preferred and one wants to minimize the number of false positives. An example of the latter case with particular biological meaning is pinpointing with great probability a region of the binding site where any docking procedure may be further explored to map all IFRs.

### Impact of including amino acid conservation descriptors on IFR classification

**Figure 4:**
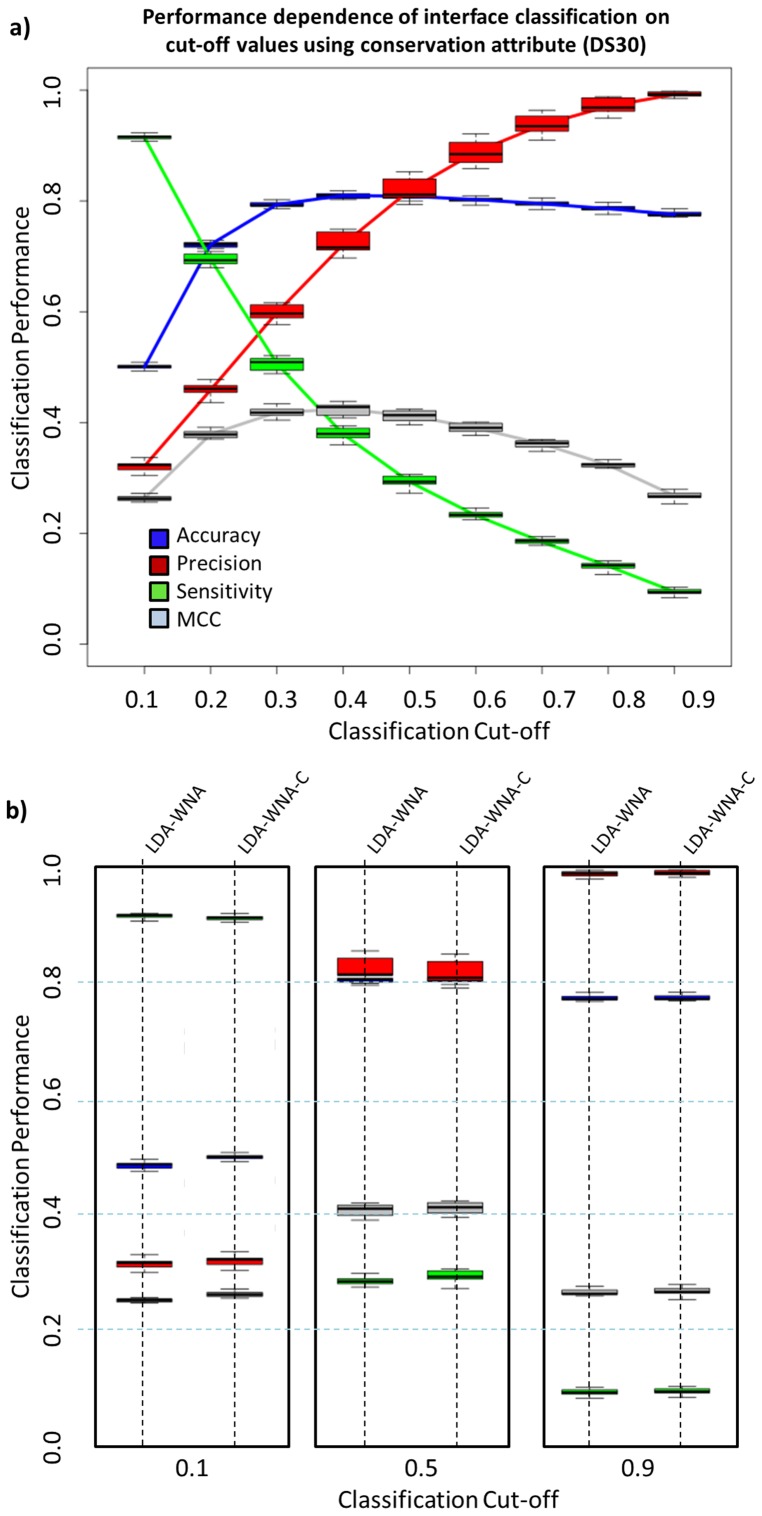
IFR prediction performance dependence on cut-off values for the LDA classifier with conservation attributes and comparison with Sting-LDA-WNA. The performance of the classifier with amino acid conservation descriptor: (a). above the classification cut-off of 0.5, the precision rate is always above 80%, reaching more than 95% with a cut-off of 0.9. The MCC rate is higher for a 0.3 cut-off; nevertheless, using a cut-off of 0.5 results in a similar MCC. (b) Comparing the performance of Sting-LDA-WNA with STING-LDA, no difference is noted for the three selected cut-off values.

To assess the completeness of the information contained in the ensemble of selected orthogonal structure attributes that were used for protein-protein interface prediction, we also included an analysis of the “*amino acid conservation*” descriptor from the STING database. The same 10-fold cross-validation scheme was maintained for LDA classifier formulation. Figure 4 shows the performance rates for this new classifier (figure 4-a) compared (for three selected classification cut-off values) with the classifier without the “*amino acid conservation*” attribute (figure 4-b). As it clearly shows, no performance gain is achieved by using amino acid conservation attributes. This result would apparently contradict the results described in the work by Liang and colleagues [22] in which they have shown that their protein-protein interface classifier (PINUP) performed better when including a conservation score. Our results indicate that all the necessary information for distinguishing IFRs from FSRs is present in the original descriptor set, (retrieved directly from a protein structure) if a sufficiently extensive list is used. Therefore, our algorithm is not eliminating the space used by other groups [55] which relay heavily on evolutionary information. In contrast, it does contemplate possibility of cases where such information is limited or even absent (orphan protein families) and where we could continue predicting contacting interface among proteins based solely on their structural and physicochemical information.

### Comparison with other methods and induced fit assessment

**Figure 5:**
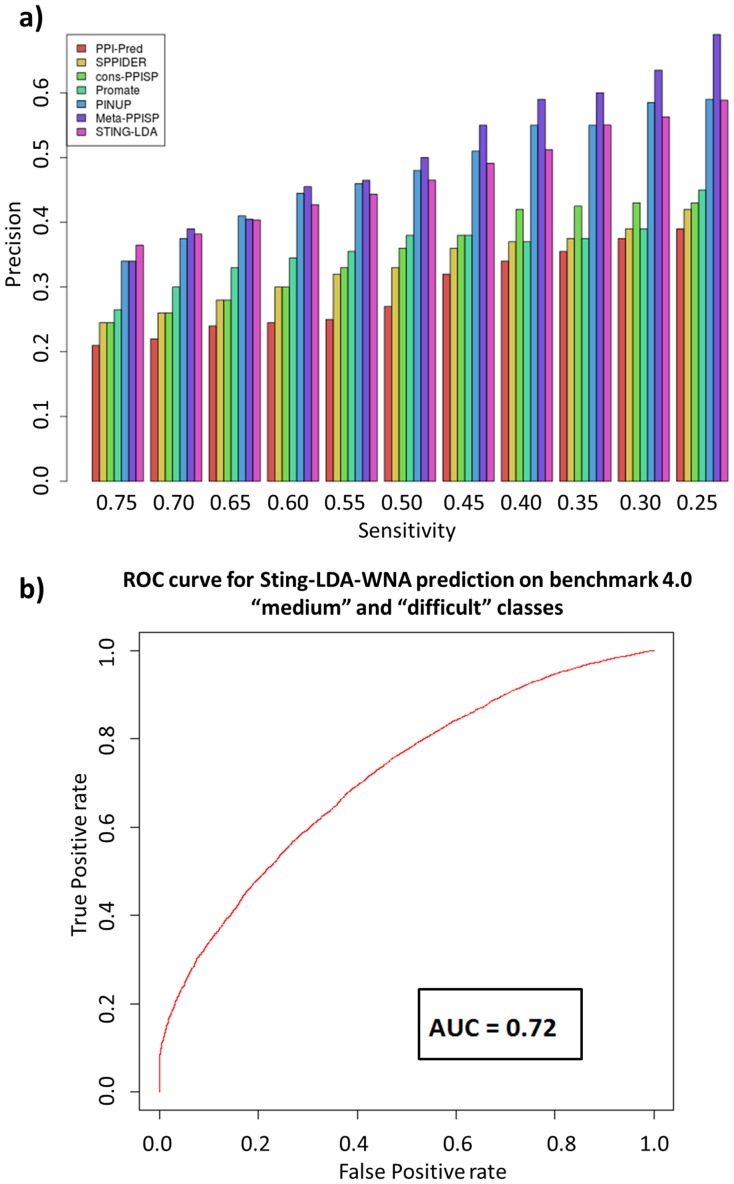
Comparison of Sting-LDA-WNA to other methods based on the test set 35Enz and induced fit assessment on benchmark 4.0 cases. (a) By selecting different thresholds for sensitivity, or coverage, the precision of the methods for IFR classification can be compared. For high interface coverage values (75%), Sting-LDA-WNA (marked in this figure as STING-LDA) has the highest precision among the used methods (37%). For balanced coverage (50%), Sting-LDA ranks third (47%), but not distant from PINUP (48%) and Meta-PPISP (50%) methods. For lower coverage (25%), Meta-PPISP still ranks first achieving 70% precision while PINUP and Sting-LDA have similar precision (59%). (b) Sting-LDA-WNA performance on the “medium” and “difficult” classes of the protein-protein docking benchmark, resulting in 6% decrease as compared to the DS30 performance, by using the AUC rate, achieving 0.72.

As noted by Porollo and Meller [23], comparisons among different methods may be limited. For instance, different methods use different binding site definitions and different training sets. Given such limitations, we tested our interface-forming residue classifier with the same test set used by Zhou and Qin [24] to compare six previously studied methods: PPI-Pred [25], SPPIDER [23], cons-PPISP [26], Promate [9], PINUP [22] and Meta-PPISP [27]. This test set is composed of 35 enzyme-inhibitor complexes (referred to as 35Enz) from the docking benchmark 2.0 [28]. For these entries, both unbound and bound structures are available. Therefore, one can also account for induced fit, i.e., the use of bound structures for prediction and unbound ones for evaluation. Figure 5-a shows the comparison of the precision rates for the listed methods using a scale of different values for sensitivity. The Sting-LDA-WNA (labeled as STING-LDA on figure 5-a) is shown to outperform almost all methods for most sensitivity cut-offs except Meta-PPISP and PINUP. In fact, the method described here has the highest precision for sensitivity of 0.75 and second highest for 0.70, 0.65 and 0.25.

Observed high precision rate at 0.25 sensitivity could be suitable for guiding docking simulations because the complete interface is extrapolated from the docking method itself. Therefore, predicting an average of 25% of the total IFRs with approximately 59% precision (similar to PINUP but lower than Meta-PPISP), which significantly reduces the number of false positive IFRs, can reduce the size of the conformation space that must be searched during successful docking procedure. At the end, the decision to what prediction cut-off to use is dependent on the user needs. For that reason, Sting-LDA returns the IFR probability for each amino acid residue.

Although the 35Enz dataset is useful for comparison of mentioned IFR prediction methods, it does not fully account for issues regarded to as “induced fit”. This is because most of the 35Enz structures are members of the so called "rigid-body" class in the protein-protein docking benchmark, i.e., they undergo very little conformational change upon protein complex formation. In order to test the IFR classifier developed in this work for cases where the protein chain undergoes conformational changes upon interface formation, we used the structures from the "medium" and "difficult" classes from completely different dataset: the protein-protein benchmark 4.0 [45]. In total, 97 protein chains were used from this new dataset to assess the limitations of our method while predicting IFRs in such scenario. Similarly to the 35Enz dataset, here we used unbound structures for prediction and bound ones for evaluating how well we ascertained the IFRs and FSRs. Also, it is important to stress that none of the structures available in 35Enz and the benchmark 4.0 were used in the training step. Figure 5-b informs that, for this particular dataset, the performance decreases by only 6% if compared to the AUC rate obtained for the DS30 dataset, showing that the method developed is robust even when significant conformational change accompanies interface formation. Unfortunately, we cannot compare the performance of other methods in that respect as the common denominator for comparison there was the use of 35Enz database (and not the benchmark 4.0). For the same reason we are unable to compare our results with those obtainable by some more recently published methods. For that, one would need to construct a new benchmark (including not only “easy” but also “medium” and “difficult” cases as well as compile all methods in a single server for posterior benchmarking. Nevertheless, a limited comparison with PredUs [52] was carried out. The authors of PredUS used the protein-protein docking benchmark for training and testing, using a 10-fold cross validation scheme. Although no Precision vs. Recall curve is shown in their report, in order to compare STING-LDA to PredUS results, following the same procedure we applied using the 35Enz dataset, the AUC rate of 0.74 in PredUS is considered. Comparing this rate to the AUC of the Sting-LDA-WNA predictor, based only on medium and difficult classes, the method described here is 0.02 lower, while using the rigid-body class, it is 0.02 higher.

The choice to use specific protein-protein docking benchmark was important so that we may have a glimpse on how robust is our method under different conditions of application. Altogether, the Sting-LDA-WNA can be used as a reliable IFR predictor for protein structures even in cases where no other homologue protein is known (eliminating demand for using conservation property in IFR prediction) and also for the scenarios where proteins undergo conformational changes while interfacing.

### Amino acid-specific classifiers: the cysteine residue example

**Figure 6:**
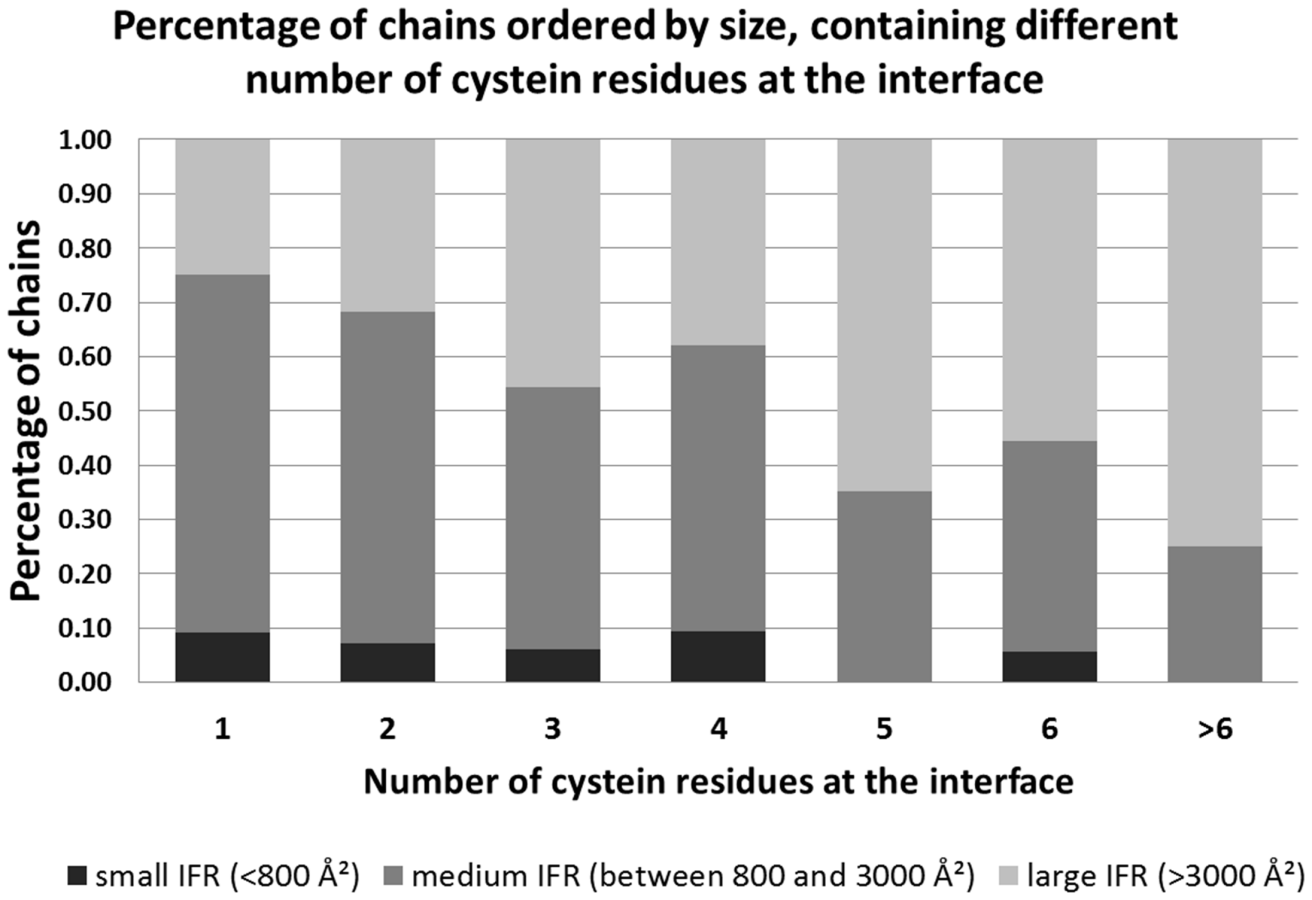
Percentage of chains (relative to the total number present in the DS35), ordered by the size of their interfaces, showing number of cysteine residues located at those interfaces. The numbers on the x-axis represent the number of cysteine residues at the interface. The interfaces are grouped in three major groups, ordered by the size. The largest number of cysteine residues is encountered at very large interfaces.

The importance of amino acid classifiers with respect to IFR localization as well as the description of local nano-environments for each IFR could be well illustrated by observing, for example, some peculiarities of cysteine residues. In another set of experiments, also concerning interface residues and their properties [29], we observed that from 6,931 chains present in DS95, 5,625 of the chains (90.15%) have cysteine residues. Additionally, the majority of these protein chains have cysteines accessible to solvent on their surface (5,201, representing 92.46% of the cysteine-containing proteins). Only half of these protein chains have cysteine residues located at an interface region, accounting for a total number of 2,653 chains (which represents 51% of 5,201 chains). We assessed the cysteine distribution in these 2,653 chains and the number of cysteines that each chain enclosed within an interface. As shown in figure 6, there is a difference in that respect between proteins with “small” interface areas and “large” interface areas. For the comparison, we defined an interface area of up to 800 Å^2^ as “small”, interface areas ranging from 800 to 3,000 Å^2^ as “medium” and the ones larger than 3,000 Å^2^ as “large” interfaces. Large interfaces appear to have a greater number of cysteine residues (as shown in figure 6), and of the 1,451 chains of this class, 789 contained cysteine residues (54.37%). This percentage for “medium” interface and “small” interface proteins is 36.2% (1,647 out of 4,549 chains) and 23.3% (217 out of 931 chains), respectively. The same analysis [29] clearly indicates that the interfaces of at least 98% of all proteins with “large” interface areas (>3000 Å^2^) are more hydrophobic than the free surface areas. This result implies that as the size of the interface grows, so does the area of hydrophobic residues that compose the selected interface and that the hydrophobic area must be buried during complex formation. At the same time, only approximately 60% of “small” interfaces (size between 200 and 500 Å^2^) are more hydrophobic than the remaining protein surface area.

Consequently, formation of protein complexes with the small interfaces might require larger stabilization (in a form of a complementary energy source to the hydrophobic interactions) to maintain their structure than the large interfaces, and it is possible that the formation of cysteine bridges is an alternative for stabilizing these interfaces. DS95 indicates that large protein interfaces contain more cysteine residues; these cysteines are clearly able to form bridges to the complementary cysteine residues located at the facing chain. However, the information stored in the STING relational database regarding all established contacts across the interfaces in DS95, clearly indicates that the “small” interfaces use a higher proportion of the available cysteine residues (in comparison with “large” interfaces), most likely for complementing the required energy for binding. For the “small” interfaces with 1, 2 or 3 cysteines, those residues form Cys bridges in at least 30% (of all DS95 identified) of the cases. In contrast, only 15% of the available cysteines are used within the “large” interface area ensemble. This finding is very important and fully supported by the data collected in the STING database. Therefore, further exploration of the details was undertaken, especially considering the specific amino acid nano-environments and condition of the broader macro-molecular environment. In addition to the role of the bonding agent between two facing interfaces, cysteine residues, located at free surfaces, can also act as components of redox sensor systems in cells. In these systems, cysteines may change their state (reduced or oxidized) according to environment status, which is influenced by the generation of reactive oxygen species and the presence of oxidized thiols [30]. When these proteins undergo changes in their structure upon oxidative stress, they can modulate cellular homeostasis, similarly to signal transducers. The presence of cysteine at interfaces is also related to stabilizing the interface itself, mainly through cysteine bridges [31]. Because cysteine residues can act as core and interface stabilizers and as redox sensors, we believe that structural and physicochemical parameters may define the nano-environment for each functional role at each specific location of a particular cysteine residue. These parameters/attributes/descriptors/features enable us to distinguish between the cysteine residues of a given chain that are located at the interface region from the ones at the free surface, as previously discussed in the Results section. This discriminative view of the residues regarding their function in a given protein is similar to the definition of the mechanisms that underlie the sensitivity of cysteines to redox status, which is related to the nano-environment wherein cysteine is located (such as its proximity to polar and charged groups [30]). Corroborating the concepts assumed in this work, this point of view indicates that physicochemical parameters define whether a region will become an interface of a protein chain upon oligomerization. Additionally, a similar approach may be applied to any type of amino acid residue with respect to its location or function and/or inclusion into a specific ensemble (IFR or FSR, as in this study, or, catalytic residue ensemble or even particular type of secondary structure element in some other studies).

## Conclusions

The importance of detecting protein-protein interactions and understanding how proteins associate with each other, giving rise to protein networks, plays a central role in systems biology. The use of structural biology tools to analyze protein networks may eventually elucidate the many possible mechanisms through which proteins can bind. This knowledge would identify important residues such as hot spots and the complementary energy sources that are eventually invoked to facilitate protein interactions. In this paper, we assessed whether the tridimensional structural information alone (without sequence conservation descriptors) can be useful in distinguishing protein binding sites from the free protein surface. The physicochemical and structural descriptors stored in the STING relational database and evaluated in this study have shown a significant discriminative power for the prediction of protein-protein interaction areas, even using a simple linear model such as the LDA. This approach is also suitable for cases (orphan proteins) in which no close homologues are known for a protein of interest because no descriptor “*amino acid sequence conservation*” (commonly employed by many previously published methods) is used.

Although we are aware of the possible data bias regarding the completeness of information for including particular amino acids into a free surface residue ensemble (knowing that no reliable information can be retrieved about real FSR), our rather simple LDA classifiers have shown high accuracy and specificity. When considering misclassifications (using the chosen 0.50 cut-off value) and using normalized values, we observed a higher number of falsely identified free surface residues than falsely identified interface forming residues. This result is of particular interest for docking algorithms because the conformation space for screening can be greatly reduced and the identification of real binding sites can be assessed using specific scoring functions, which is yet to be accomplished and will be studied in our future work.

ROC analysis has shown that our method performs much better than a random classifier, even for the worst specific case: the glycine LDA classifier. The Sting-LDA-WNA classifier results for the 20 amino acid-specific LDA classifiers were evaluated using the Matthew’s correlation coefficient and AUC criteria. From these tests, we found that the Sting-LDA-WNA classifier had significant discriminative power, mainly for amino acids such as tryptophan, aspartic acid, methionine, isoleucine, leucine and valine. In particular, we showed a significant gain in IFR prediction performance when dividing the dataset (DS30) into the 20 datamarts corresponding to the 20 amino acids. We showed that when using the same data and the same model (LDA), IFRs can be better predicted using specific amino acid classifiers than when no such distinction is used. To our knowledge, this is the first time that such an approach has been performed, and it may be extended to other existing methods for improved performance. The motivation for creating amino acid-specific classifiers arises from the fact that we found a different number of principal components (orthogonal attributes) for each of the 20 amino acids. Also, we show, by using the “medium” and “difficult” classes of the protein-protein docking benchmark 4.0, that the Sting-LDA-WNA classifier is fairly robust regarding its application in scenarios where some conformational change accompanies protein complex formation.

The resulting procedure is now implemented in the BlueStar STING suite of software (http://www.cbi.cnptia.embrapa.br/SMS/predictions/index.html) and can be used for predicting interface residues for all current entries in the PDB.

## Methods

### Dataset Selection

**Figure 7:**
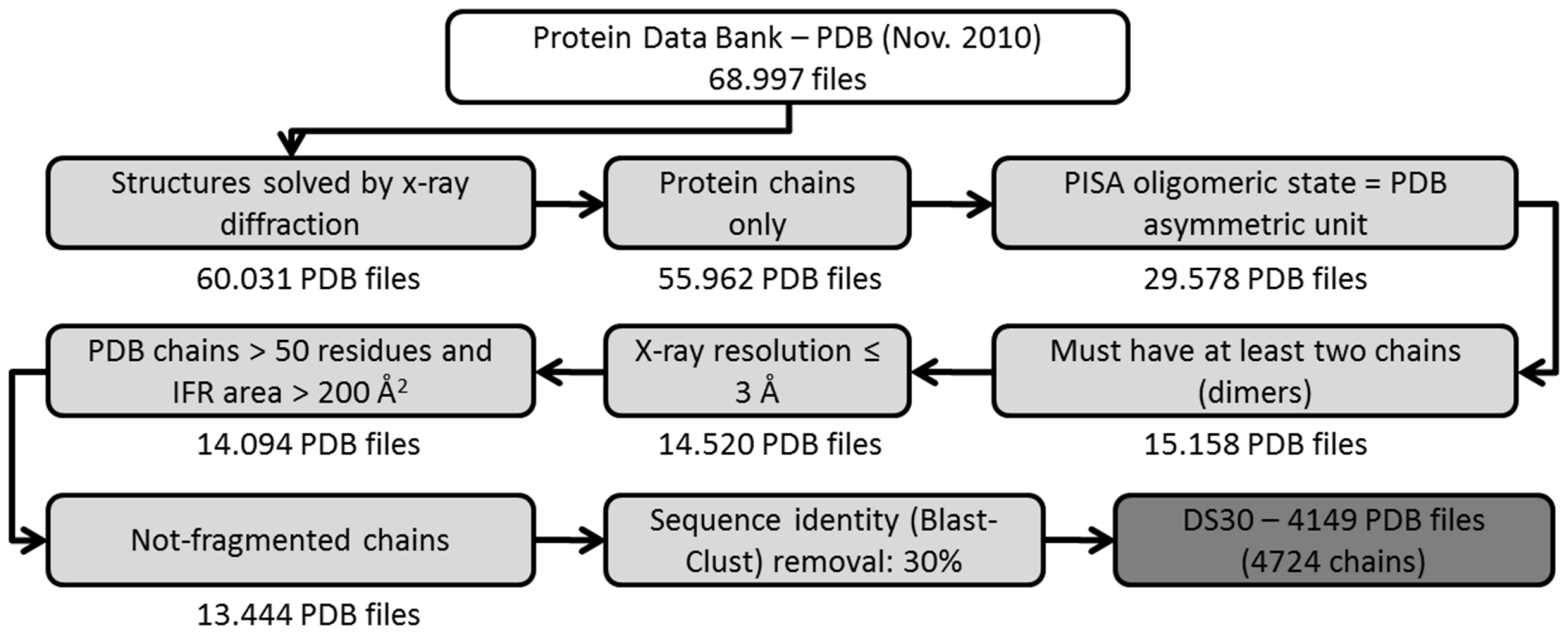
Filters used to establish DS30. Filters were used in sequential order to eliminate the structures accessed from three databases (PDB, PISA and UniProt) and to select the protein complexes used for this study. In November 2010, there were 68,997 structures in the PDB, and 60,031 of those entries were solved using x-ray crystallography. Removing DNA/RNA chains resulted in 55,962 files. Only 29,578 entries had an oligomeric state as defined by a PISA equal to the PDB asymmetric unit. Approximately half of these entries were found to have at least two chains. We defined a crystal resolution threshold, and only used structures that were solved with resolutions up to 3 Å. The double filter removed chains smaller than 50 residues and interface areas smaller than 200 Å^2^, yielding 14,094 files. A few entries were removed because for having a fragment flag in the UniProt database. The last filter removed entries with 30% positional sequence identity. The resulting datasets are referred to as DS30 (4,219 PDB files).

Three-dimensional structures were selected from the Protein Data Bank [32] to analyze the protein-protein complexes in this study. A set of filters (figure 7) was used to select only those structures that had an equal number of chains in their asymmetric unit and also in an oligomeric state as defined by the PISA database (*Protein Interfaces, Surfaces and Assemblies*) [33]. According to Xu *et al.* (2008) [34], the PISA oligomeric state is the most accurate for protein complexes compared with the PDB and PQS (which is now out of date). This filter makes it more likely to retrieve only biologically relevant complexes, excluding possible crystal complexes (artificially formed macromolecular complexes resulting from the requirements imposed by the crystalline state that have no relevant biological importance or activity).

Additional data filtration prevented those proteins that are assigned as "fragment" by UniProtKB from consideration in this work [35]. The PDBSWS - PDB/UniProt Mapping database was used to include identifiers of UniProtKB into the PDB [36] and then perform the required filtration.

According to Scheneider *et al.* [37], there are significant differences between protein structures solved using x-ray crystallography and those solved using nuclear magnetic resonance (NMR), especially with respect to the type and number of contacts between amino acid residues. For that reason, we only selected x-ray complexes. Our decision was also guided by the far fewer number of protein-protein complexes solved by NMR (because of the macromolecular size limitation of this technique). Crystal complexes (biologically irrelevant) have a relatively small interface area [7]. Thus, those complexes with interface areas of less than 200 Å^2^ were also excluded. The complexes formed by proteins with other macromolecules, such as DNA and/or RNA, were also excluded from our dataset.

The final step in data filtration was removing redundancy from the protein sequences using a 30% cut-off value (alternative values of 70 and 95% were also employed, producing datasets DS70 and DS95, respectively, for comparative studies). This procedure removes all chains built from primary sequences that have similarities higher than 30%. To guarantee the removal of redundant data (therefore avoiding model over-fitting), no single chain is present more than once in the DS30, even if it makes more than one distinct interface with different pairing chains.

It is important to mention here that we made, in this work, no distinction regarding homo- and hetero-complexes (neither for training nor testing). As shown by Chen and Zhou [26], no clear advantage arises from using solely hetero-mers for training and evaluation. Actually, the training being performed by using all complexes and testing on hetero-complexes gave slightly better results (“strict” accuracy shown in table II from Chen and Zhou, 2005).

Additionally, we found that the composition of the DS30 is as following: the total number of homo complexes accounts for 69.33% and hetero-complexes, for 30.67% of the total (as established after consulting corresponding PISA information). The list of all chains with regard of this specific classification is also provided at our server web page.As previously mentioned, including IFRs from alternative partners partially and indirectly compensates for not mapping alternative IFRs on the “base” structure (where the “base” structure is the one that forms more than one interface with different partners). With this method, data redundancy is also completely avoided. Because of some missing attributes in the BlueStar STING database, we had to discard another 121 chains. The resulting dataset, referred to as DS30, contains 4149 non-redundant PDB files. Each PDB file from DS30 was further divided, by randomly selecting structures, into 10 groups to perform 10-fold cross validation.

### Physicochemical and structural parameters/descriptors/attributes

The STING database covers all the proteins in the PDB, which is updated weekly. STING has over 1050 descriptors that are provided in a residue-by-residue manner (most of which are calculated using homemade algorithms) and divided into 97 different classes. Approximate calculations suggest that STING is the largest databank of its type. Each PDB entry has in an average of 2.68 chains and each chain has an average of 222 residues. Therefore, the STING database has over 5.3×10^10^ descriptor records.

In this study, we consider only a fraction of all of the descriptors in the STING database (table 1). The descriptors were organized into a relational database, allowing multiple (usually similar and/or homologous) structures to be analyzed at the same time. The full definition of the descriptors used in this work is not provided here (being beyond the scope of this work). However, a very short outline follows to make reading easier. Alternatively, readers are invited to access the full descriptions published previously on STING’s web-server site at http://www.cbi.cnptia.embrapa.br/SMS/ STINGm/help/MegaHelp_JPD.html#chainsparameters [38] and in several papers [10]–[13], [20].

A short description of each used attribute follows:

**Accessibility [Acc].** BlueStar STING uses the rolling sphere algorithm (Lee e Richards, 1971) to calculate protein surface accessibility. BlueStar STING uses the software *SurfV* [39] to calculate the accessibility of amino acids to a solvent. For protein complexes, *SurfV* calculates the amino acid-accessible surface for each individual chain (*accessibility in isolation*) and for the complete PDB (*accessibility in complex*). The amino acids that undergo a change in their accessibility upon protein-protein complex formation are referred to as IFRs. Their corresponding (interface) area is the difference between *accessibility in isolation* and *accessibility in complex.*

**Electrostatic Potential [EP].** BlueStar STING uses a modified version of DelPhi software for high-throughput calculation (Rocchia e Neshich, 2007). DelPhi solves the Poisson-Boltzmann equation for each group of fixed charge points that constitutes each type of amino acid in a protein.

**Hydrophobicity.** BlueStar STING uses the Radzicka amino acid scale of hydrophobicity weighted by the amino acid relative accessibility as a definition of amino acid hydrophobicity.

**Cross-Link Order [CLO].** This attribute is defined as the number of amino acids undergoing any type of interaction (any one (or combination) of 6 possible types of interatomic contacts) that are separated by at least 30 amino acids in sequence but close in three-dimensional structure.

**Cross-Presence Order [CPO].** This attribute is defined as the number of amino acids that are separated by at least 30 amino acids in sequence but close in the three-dimensional structure within a probe sphere of radius 8.0 Å.

**Dihedral Angles.** The PHI and PSI angle of the main chain.

**Side Chain Angles.** The CHI 1 to CHI 4 angles of the amino acid side-chains. These angles are not suitable for calculation for all the amino acid types (e.g. glycine).

**Density.** A probe sphere of varying radius (from 3 to 7 Å) is centered either on the carbon alpha (CA) or the last heavy atom (LHA) in the amino acid side chain. The masses of the atoms are summed and divided by the probing sphere volume.

**Sponge.** This attribute is similar to the density definition in calculation, but instead of using the atomic masses, the volumes of the atoms are subtracted from the probing sphere volume and normalized.

**Contact Energies.** Atoms or residue pairs are associated with the following energy values: hydrophobic interaction  =  0.6 kcal/mol; aromatic stacking  =  1.5 kcal/mol; hydrogen bond  =  2.6 kcal/mol; salt bridge  =  10.0 kcal/mol; and cysteine bridge  =  85.0 kcal/mol.

**Unused Contact Energies.** The entire PDB is screened for the maximum contact energy of each amino acid type. This maximum value is subtracted from the contact energy of each specific amino acid. The resulting value is considered the amino acid contact potential, which is yet to be established.

Following the definition of weighted neighbor averages (WNA) as described by Porollo and Meller [23], each used descriptor *D* was averaged for neighboring amino acid values *D_i_* using two distinct equations:
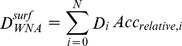
(1)

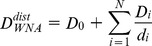
(2)where *N* is the number of neighbors within 15 Å from the amino acid of interest, *Acc_relative,i_* is the relative accessibility, and *d_i_* is the distance of the i-th amino acid from the amino acid of interest. The reason for selecting the cut-off value of 15 Å is justified in da Silveira et al. [44] by considering the largest distance from which contacting residues still influence the central residue.

### Linear correlation among variables

We checked for the possible existence of linearly correlated attribute variables among the same attribute class. In a second step, the linear correlations among different attributes were removed by principal component analysis (as detailed in the next section). We employed the *linear correlation coefficient* (*r*) defined for pairs of variables *x* and *y* as follows:
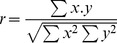
(3)


A threshold of 0.85 (highly correlated variables) was used for *r*. The variable (and attribute) most correlated to others was removed from consideration in the forthcoming construction of multivariate linear classifiers for IFR prediction.

### Principal component analysis

As an alternative to using all of the remaining descriptors as inputs for the linear classifiers, we performed principal component analysis and calculated the principal component scores for all of the amino acids. Principal components form a set of normally distributed and orthogonal variables (linear correlation coefficient vanishes for each pair of principal components). Both characteristics are assumed when performing LDA analysis. The correlation matrix was calculated using only the training set, and the generated principal components were applied to both the training set and the test set. It is important to emphasize that the resulting principal components are all orthogonal and normally distributed. There are as many principal components as there are variables in that new dataset. We defined the number of used principal components as the minimal number that can account for 95% of the total variability in the original training set [40]. This procedure was repeated for 10-fold cross validation. Additionally, we divided the training set and the test set according to amino acid type. In total, we have analyzed 200 classifiers (20 amino acid-specific classifiers for each of the 10-fold cross validation group).

### LDA classifiers

The LDA methodology uses a training set to calculate the mean and standard deviation values for each attribute and thus discriminate between the two groups: IFR and FSR. With these values, LDA uses maximum likelihood estimation with a Gaussian probability distribution to determine the a posteriori probabilities for each amino acid from the test set [40].

### Evaluation of LDA classifiers

Using linear discriminant analysis [40], [41], we linearly combined the new set of attributes and their variables (principal components). This procedure resulted in 20 classifiers (one for each amino acid type). Each amino acid-specific classifier was applied to its respective test set. As far as we know, this is the first time that amino acid-specific classifiers have been used for IFR prediction. To measure the performance of the generated classifiers with the test sets, we used ROC analysis [42], [43] and two criteria: the area under the ROC curve (AUC) and the maximum Matthew’s correlation coefficient (MCC) defined by:
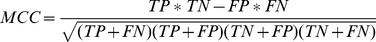
(4)where TP, TN, FP and FN represent true positive, true negative, false positive and false negative, respectively.

The MCC rate is defined for algorithms used for binary classification between two classes (in this work: interface forming residues and free surface residues). MCC ranges from -1 to +1, and classifiers with better discriminative power have higher MCC values. For a random classifier, the MCC rate is equal to zero. For each cut-off used to plot the ROC curve, we calculated the MCC rate and the maximum MCC that can be used to choose a specific classification cut-off.

Following, to establish the final classifier, we simply combined the results of the 20 amino acid-specific classifiers into a single classification file. As shown, the division and further summation of the 20 amino acid prediction classifiers improved the performance. LDA classifiers can determine a posteriori probabilities for whether each amino acid will be classified into the IFR or FSR ensemble. The performance change was calculated (using MCC and the three other rates described below) for different cut-offs for this probability.

Different methods for predicting protein-protein interfaces, cited in the literature, use different performance measures. Therefore, to compare our predictor to others, the following three rates were calculated:
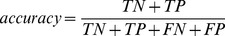
(5)

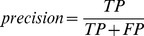
(6)

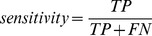
(7)


Accuracy is a general performance measurement that does not differentiate between *true positives* and *true negatives*, as accuracy is a somewhat simplistic rate. Precision is related to how confident we are when predicting that a given amino acid is an IFR. With higher precision, fewer *false positives* are found in the prediction. Sensitivity gives an idea of the coverage of the interface being predicted. When sensitivity values are higher, fewer *false negatives* occur in the prediction. Following the procedure of Zhou and Qin [24], a direct comparison to other methods was performed using precision-sensitivity values. Therefore, a specific value of coverage (sensitivity) may be selected, and the respective precision values are then compared.
